# 

**Published:** 1917-05-05

**Authors:** 


### CONTENTS.

PAGE

**The Medical Profession and the War Office** ... 79

**Optimism and Pessimism** ... ... ... ... ... 80

**Hospital and Institutional News** ... ... ... 81

King Manoel Inspects a Liverpool War Institution—A Friend at Court—Charging Hospital Patients for Drugs—Important to Residents in London—The Prince of Wales' Hospital, Cardiff, for Limbless Sailors and Soldiers—Graceful Gift to King Edward VII.'s Hospital, Cardiff—More Bequests for Glasgow Hospitals—Endowments for the Sick Poor in Convalescence—The Widow's Mite—The Latest Hospital History—The Partition of an Infirmary Chaplaincy—A Trench Exhibition—The Work of the London Hampshire Society—After the Explosion—A Careful Record of Red-Cross Work—Criticism of the Ecclesiastical Commissioners—Rations in V.A.D. Hospitals—A Medical Man on Rations in Hospitals—The M.A.B. and the Diet Scale of Its Employees—Indian Children and Infant Foods—Chairmen Who " Fire " Hospital Meetings—The Creche and the Working Mother—One Health Visitor for 500 Births—The Case for Mothers' Pensions—An Attendant Evil of Social Reform—A Municipal Hospital—Rowdyism at Soldiers' Concerts—The April *Searchlight*—Pharmacists Help the Great Northern Hospital—This Week's Drug Market.

PAGE

**A Medical Aspect of the Education Proposals** 87

By a Tuberculosis Worker.

**Plastic Surgery** ... ... ... ... ... ... ... 88

**King Edward's Hospital Fund for London** ... 89

Annual Meeting of Governors and General Council.

**The Institutional Library** ... ... ... ... 91

**The Matrons' and Sisters' Department** ... 93

The Nation's Fund for Nurses.

Round the Hospitals.

Facts Destroy False Criticism—Mr. Stanley at Queen's Hall—The Scottish and Irish College Boards—Proposed Diploma for Irish Nurses—The Army's Demand for Nurses—A Great Opportunity for the P.L.I.M.A.

**The Royal College of British Nursing** ... ... 95

The Great Concert and Two Important Speeches.

**The College of Nursing, Limited** ... ... ... 97

**The Editor's Letter-Box** ... ... ... ... 97

**Food in Wartime** ... ... ... ... ... ... 98

The Problem of Substitutes.

**Some Problems of To-day** ... ... ... ... 99

III. The Parent: Supported or Supplanted.

**Matrons' and Sisters' Appointments** ... ... 100

**The Institutional Worker** ... ... (Suppl.) 1

Electro-Medical Apparatus.

